# Citicoline and Memory Function in Healthy Older Adults: A Randomized, Double-Blind, Placebo-Controlled Clinical Trial

**DOI:** 10.1093/jn/nxab119

**Published:** 2021-05-12

**Authors:** Eri Nakazaki, Eunice Mah, Kristen Sanoshy, Danielle Citrolo, Fumiko Watanabe

**Affiliations:** Research & Innovation Center, Kyowa Hakko Bio Co., Ltd, Tsukuba, Ibaraki, 305–0841, Japan; Biofortis Innovation Services, Addison, IL 60101, USA; Biofortis Innovation Services, Addison, IL 60101, USA; Scientific and Regulatory Affairs, Kyowa Hakko USA Inc., New York, NY 10016, USA; Research & Innovation Center, Kyowa Hakko Bio Co., Ltd, Tsukuba, Ibaraki, 305–0841, Japan

**Keywords:** 5′-cytidine diphosphate choline, citicoline, aging, brain, memory loss

## Abstract

**Background:**

Supplementation of citicoline (CDP-choline), a naturally occurring mononucleotide, has shown beneficial effects on memory function and behavior in populations with a wide range of impairments. However, few studies have investigated its effect in healthy older populations.

**Objective:**

The objective of this study was to investigate the effects of citicoline (Cognizin^®^), on memory in healthy elderly populations with age-associated memory impairment (AAMI).

**Methods:**

A total of 100 healthy men and women aged between 50 and 85 y with AAMI participated in this randomized, double-blind, placebo-controlled trial. Participants were randomized to receive placebo (*n* = 51) or citicoline (*n* = 49; 500 mg/d) for 12 wk. Memory function was assessed at baseline and end of the intervention (12 wk) using computerized tests (Cambridge Brain Sciences, Ontario, Canada). Safety measurements included adverse events query, body weight, blood pressure, and hematology and metabolic panel. Intent-to-treat analysis was conducted using ANCOVA for the primary and secondary outcome variables with Bonferroni correction for multiple comparisons.

**Results:**

A total of 99 out of 100 participants completed the study in its entirety. After the 12-wk intervention, participants supplemented with citicoline showed significantly greater improvements in secondary outcomes of episodic memory (assessed by the Paired Associate test), compared with those on placebo (mean: 0.15 vs. 0.06, respectively, *P* = 0.0025). Composite memory (secondary outcome), calculated using the scores of 4 memory tests, also significantly improved to a greater extent following citicoline supplementation (mean: 3.78) compared with placebo (mean: 0.72, *P* = 0.0052).

**Conclusions:**

Dietary supplementation of citicoline for 12 wk improved overall memory performance, especially episodic memory, in healthy older males and females with AAMI. The findings suggest that regular consumption of citicoline may be safe and potentially beneficial against memory loss due to aging. This trial was registered at clinicaltrials.gov as NCT03369925.

## Introduction

The world's population is aging with almost every country experiencing a growth in the number and proportion of older persons. Projections of the World Population Prospects by the United Nations suggest that 1 in 6 people (16%) in the world will be aged over 65 y by 2050 (1). An expected challenge of an aging population is the increased rates of health issues associated with aging, such as age-related cognitive decline. Although age is the most important predictor of cognitive decline, this process is a complex interplay of many contributing factors including demographic, genetic, socioeconomic, environmental, and nutritional parameters (2). No effective pharmaceutical treatments for age-related cognitive decline are available, emphasizing the importance of prevention strategies against cognitive decline. There is extensive evidence on how nutrients and bioactive compounds may impact on cognitive decline due to aging (3). Some evidence points to specific dietary patterns (e.g., ketogenic or Mediterranean diets) as having strong potential to combat age-related cognitive decline (4). Additionally, individual nutrients such as B vitamins, antioxidant polyphenols, selenium, vitamin D, medium-chain triglycerides, and long-chain omega-3 fatty acids have been investigated for their potential cognitive-enhancing properties (5).

An important nutrient that is known to affect brain development and aging is choline. Choline is essential for several biological functions of cells. For example, choline metabolites acetylcholine and phosphatidylcholine, respectively, contribute to proper signaling functions for normal cholinergic neurotransmission and the structural integrity of cell membranes (6). Brain choline uptake is decreased in older adults (7) and the lower supply of extracellular choline may contribute to aging-related cognitive decline. Not surprisingly, higher dietary choline is associated with better cognitive performance in elderly individuals (8, 9). One method to increase dietary choline is through supplementation with citicoline (CDP-choline or cytidine-5′-diphosphate choline), choline salts, such as choline chloride and choline bitartrate, glycerophosphocholine, as well as phosphatidylcholine. Compared with choline moiety obtained through other dietary sources such as phosphatidylcholine, choline in citicoline has been suggested to be less prone to conversion to trimethylamine (TMA) and trimethylamine N-oxide (TMAO), which have been implicated in the pathogenesis of cardiovascular disease (10). Citicoline is a naturally occurring mononucleotide comprised of cytosine, ribose, pyrophosphate, and choline. It is produced by the body as an intermediate product of phosphatidylcholine and sphingomyelin biosynthesis (11, 12). The neuroprotective actions of citicoline include activating the biosynthesis of structural phospholipids in the neuronal membranes, increasing cerebral metabolism, noradrenaline, and dopamine levels in the central nervous system, preventing the loss of cardiolipin (an exclusive inner mitochondrial phospholipid enriched with unsaturated fatty acids), and protecting cell membranes by accelerating re-synthesis of phospholipids (13). Unsurprisingly, citicoline supplementation has shown beneficial effects on memory function and behavior in populations with a wide range of impairments such as those with mild to moderate vascular cognitive impairment, vascular dementia, or senile dementia (14).

In healthy populations, oral intake of a citicoline supplement (Cognizin), improved attention compared with placebo in middle-aged women [250 mg/d for 1 mo, age 40–60 y (15)] and in adolescent males [250 and 500 mg/d for 28 d, *n* = 24/group (16)]. Additionally, oral citicoline supplementation (1 g/d for 3 mo) improved logical memory score compared with placebo in men and women (*n* = 49/group, age 50–85 y) with relatively inefficient memory [i.e., scored below average of all recruited participants (17)]. Another open-label clinical trial demonstrated a significant improvement in word and object recalls after citicoline supplementation (1 g/d) for 28 d (18). To date, no studies have investigated the effects of citicoline supplementation at 500 mg/d on memory in healthy elderly adults with age-associated memory impairment (AAMI). Thus, the objective of this study was to investigate the effects of citicoline supplementation at 500 mg/d on memory in healthy elderly populations with AAMI.

## Methods

### Study design

This randomized, double-blind, placebo-controlled trial of citicoline was conducted at Biofortis Inc. (Addison, IL) between January 2018 and December 2018. This study was carried out in compliance with the protocol and in accordance with Good Clinical Practices (GCP), the applicable US Code of Federal Regulations (CFR), and the Declaration of Helsinki (2013 Version). The study protocol was approved by an Institutional Review Board (IntegReview, Austin, TX). Signed written informed consent for participation in the study was obtained from all participants before protocol-specific procedures were carried out. This trial was registered at ClinicalTrials.gov with identifier NCT03369925.

### Study participants

Healthy men and women (aged 50 to 85 y) with AAMI were recruited by the Biofortis Clinical Research team by using an established database of volunteers and local advertisements. While there is no agreed-upon definition for AAMI, we based our recruitment on the criteria for AAMI suggested by the US National Institutes of Health (19). Thus, the inclusion criteria for this study included: age 50–85 y, scored ≥24 on the Mini-Mental State Examination, ≥85 on the Kaufman Brief Intelligence Test - Second Edition, ≤5 on the Geriatric Depression Scale, and 4, 3, or 2 on the Spatial Span test, and no health conditions that would prevent him or her from fulfilling the study requirements on the basis of medical history and routine laboratory test results. Exclusion criteria included color blindness, abnormal laboratory test results that fell outside of the normal range as defined by the analytical laboratory (Elmhurst Memorial Reference Laboratory, Elmhurst, IL), major medical or neurological illness including, but not limited to, hyperparathyroidism, type 1 or 2 diabetes mellitus, hypoglycemia, myocardial infarction, peripheral arterial disease, uncontrolled asthma, Alzheimer's disease, Parkinson's disease, stroke, intracranial hemorrhage, and local brain lesions, females who were pregnant or planning to be pregnant during the study period, and medications that may have interfered with the interpretation of the study results (e.g., medications known to affect cognition).

### Study product and treatment

Eligible participants were randomly assigned 1:1 to oral citicoline (500 mg/d) or placebo in a double-blind design using a randomization sequence prepared by the lead study statistician. The randomization sequence was designed such that it allowed approximately equal distribution of baseline spatial span test score and sex among the 2 test groups. A randomization number was assigned to eligible participants using an electronic randomization module (Medrio, Inc., San Francisco, CA). Participants, research staff, and outcome assessors were blinded to group allocations until data analyses had been completed.

Study supplements consisted of encapsulated microcrystalline cellulose (placebo), or 250 mg/capsule of citicoline (Cognizin; Kyowa Hakko Bio Co., Ltd). Placebo and citicoline capsules were identical in color and size. Participants were instructed to consume 2 capsules with breakfast for 12 wk; thus participants in the citicoline group consumed a total of 500 mg/d of citicoline. Compliance was documented as a percentage of study product consumed calculated based on scheduled product intakes and number of returned study product. Non-compliance was defined as consumption of <80% or >120% of the scheduled intake upon study completion. Overall compliance was determined at Week 12.

### Study procedures

This study consisted of 1 screening visit, 2 test visits (Week 0 and Week 12), and 1 compliance visit (Week 6). To minimize the impact of lifestyle changes on cognition, participants were instructed to maintain their habitual diet, exercise routines, and sleep duration throughout the study and any major change/life stress event that could impact cognition was inquired and documented. At screening, participants were screened for inclusion and exclusion criteria. Additionally, evaluations of medical history, prior/current medication/supplement use, height, body weight measured using a digital scale (Health-O-Meter 349KLX; Sunbeam Products, Inc., Boca Raton, FL), vital signs including systolic and diastolic BP measured using an automated blood pressure monitor (Welch Allyn 53000; Hill-Rom Holdings, Inc., Chicago, IL), Mini-Mental State Examination, Kaufman Brief Intelligence Test - Second Edition, Geriatric Depression Scale, and sleep and smoking habits were assessed. The 24-h diet record was collected and reviewed to compare food and beverage consumption up to the day before test visits (Weeks 0 and 12) for consistency. BMI was calculated as kg/m^2^. At baseline (Week 0) and end of study (Week 12), participants arrived at the clinic fasted (10–14 h), consumed a standard breakfast, and completed the cognitive assessments test battery. Fasting (10–14 h) blood samples were collected at the screening and end of study visits for hematology and metabolic panel assessments.

### Cognitive assessments

Cognitive performance was assessed using Cambridge Brain Sciences (Toronto, Ontario, Canada) computerized tests. This computerized testing battery was publicly available and was validated by the Medical Research Council and Brain Sciences Unit [Cambridge, UK (20)]. The testing battery was used to assess working memory (Monkey Ladder task), short-term spatial memory (Spatial Span), short-term verbal memory (Digit Span task), episodic memory (Paired Associate task), selective attention (Feature Match and Interlocking Polygons tasks), and sustained attention (Sustained Attention to Response Task). Normative data from >74,000 participants aged between 11 and 100 y provided by Cambridge Brain Sciences showed a decrease in Spatial Span score with age. Thus, it was used at screening to identify participants with compromised memory function defined as scoring at least 1 standard deviation below the mean established for young adults (based on normative data provided by Cambridge Brain Sciences). Additionally, screening Spatial Span scores were also used during randomization whereby participants were stratified into 3 Spatial Span test score strata (score of 2, 3, or 4). All 7 tasks were administered at Week 0 and Week 12, but not administered at Week 6. Participants were given the opportunity to practice all cognitive tasks during the screening visit.

### Safety assessments

Safety was assessed by adverse events (AEs) reported by participants, as well as assessment of vital signs, body weight, and hematology and metabolic panels. Inquiry of AEs was conducted using an open-ended question at Weeks 0, 6, and 12, and during phone calls between each visit. The Clinical Investigator evaluated all AEs with respect to their severity, according to the World Health Organization Adverse Reaction Terminology (WHO-ART) dictionary (21). The Clinical Investigator also judged the likelihood that the AE was related to the study product in accordance with Reviewer Guidance on Conducting a Clinical Safety Review of a New Product Application and Preparing a Report on the Review (22). Finally, metabolic and hematology panels were evaluated by Elmhurst Memorial Reference Laboratory (Elmhurst, IL) from heparin plasma samples using the Dimension Vista 500 System (Siemens Medical Solutions USA, Inc., Malvern, PA) and from whole blood samples using the Sysmex XN-3100 Automated Hematology System (Sysmex America, Inc., Lincolnshire, IL), respectively.

### Statistical analysis

Sample size calculations were performed using G*Power (Version 3.1.9.2), for ANCOVA (fixed effects, main effects, and interactions) with the following parameters: α err prob = 0.05, power (1-β err prob) = 0.80, numerator df = 1, number of groups = 2, number of covariates = 2. Sample size calculations also used proprietary normative data (obtained from >74,000 adults) for Spatial Span provided by Cambridge Brain Sciences and estimated improvement difference of 9.9% and distribution in scores following citicoline supplementation between groups based on using data from a previous study on citicoline and memory (17), while taking into account 2 covariates (age and baseline scores). An evaluable sample size of 82 was needed to detect a significant difference between groups and a total of 100 participants were randomized to account for possible attrition.

The primary outcome variable was the raw change in Spatial Span scores, calculated as the difference in baseline score and end of the test period (Week 12) score for each participant. Secondary outcome variables included the raw change in test scores for the remaining 6 cognitive tasks. Additionally, the composite memory score was calculated for overall memory function based on the Spatial Span (SS_score_), Monkey Ladder (ML_score_), Paired Associates (PA_score_), and Digit Span (DS_score_) raw scores using the following formula provided by Cambridge Brain Sciences:

$$\begin{eqnarray*}
CompositeMemoryScore = \left( {\tilde{Q}\cdot 15} \right) + 100
\end{eqnarray*}$$

*where*

$$\begin{eqnarray*}
{\tilde{Q}} = \frac{{\left[ {\left\{ {\left( {\frac{{S{S_{score}} - 6.11}}{{1.07}}} \right)\cdot \ 0.69} \right\} + \left\{ {\left( {\frac{{M{L_{score}} - 7.8}}{{1.16}}} \right)\cdot \ 0.69} \right\} + \left\{ {\left( {\frac{{P{A_{score}} - 5.24}}{{1.11}}} \right)\cdot \ 0.58} \right\} + \left\{ {\left( {\frac{{D{S_{score}} - 7.15}}{{1.48}}} \right)\cdot \ 0.26} \right\}} \right]}}{{\left( {4\cdot \ 0.39} \right)}}
\end{eqnarray*}$$

All statistical analyses were conducted using SAS for Windows (version 9.4, Cary, NC) on the intent-to-treat (ITT) population, which included all participants who were randomized into the study. All tests of significance, unless otherwise stated, were performed at alpha = 0.00625 (2-sided), which accounted for multiple comparisons with Bonferroni correction. ANCOVA was used to assess differences between test groups for the primary and secondary outcome variables. Initial ANCOVA models contained terms for test group, Spatial Span screening score (i.e., 4, 3, 2), sex, BMI, 2-way interaction terms “test group by sex,” “test group by second Spatial Span screening score,” “test group by BMI,” and “test group by age,” with age and baseline test scores as covariates. Models were reduced using a backward selection method until only terms for test group, Spatial Span screening score (i.e., 4, 3, 2), sex, age, baseline test scores, and significant 2-way interaction terms (if there are any) remained in the model. Data of unadjusted means ± SEM were presented for each test group. Assumption of normality of residuals was investigated for each outcome variable at the 5% level of significance with the Shapiro-Wilk test (23).

For hematology and metabolic panels, continuous measures were compared between groups with the Wilcoxon rank sum test. At each time point, the *P* values were adjusted for multiple comparisons with the false discovery rate. Data of unadjusted means ± SD are presented for each test group.

Finally, differences between groups for baseline characteristics (**Table 1**), were analyzed using independent *t*-test for continuous variables and Fisher's exact for categorical variables.

**TABLE 1:** Characteristics of the 100 study participants enrolled in this randomized controlled trial^1^

	Placebo	Citicoline	
	(*n* = 51)	(*n* = 49)	*P*
Age,^2^ y	65.5 ± 1.13	63.2 ± 1.12	0.53
Gender,^3^ *n* (%)			
Male	16 (31.4)	19 (38.8)	
Female	35 (68.6)	30 (61.2)	0.16
Race,^3^*n* (%)			0.17
White	47 (92.2)	41 (83.7)	
Black/African American	3 (5.88)	7 (14.3)	
American Indian or Alaskan Native	0 (0.00)	1 (2.04)	
Asian	0 (0.00)	0 (0.00)	
Native Hawaiian or other Pacific Islander	1 (1.92)	0 (0.00)	
Ethnicity,^3^*n* (%)			1.00
Not Hispanic/Latino	49 (96.1)	48 (98.0)	
Hispanic/Latino	2 (3.92)	1 (2.04)	
BMI,^2^ kg/m^2^	29.6 ± 0.96	30.7 ± 0.91	0.35
Systolic BP,^2^ mm Hg	127 ± 2.06	130 ± 1.83	0.25
Diastolic BP,^2^ mm Hg	74.4 ± 1.64	76.1 ± 1.38	0.44
MMSE^2^	27.6 ± 0.22	27.8 ± 0.21	0.50
KBIT-2^2^	101 ± 1.45	98.9 ± 1.26	0.30
Geriatric Depression Scale^2^	0.784 ± 0.144	0.796 ± 0.124	0.95
Spatial Span score (at screening)^2^	3.71 ± 0.08	3.80 ± 0.06	0.35
Hours of sleep (at Week 0),^2^ h	7.32 ± 0.12	7.20 ± 0.11	0.47

1 Values are means ± SEMs or frequency (%). Differences between placebo and citicoline groups for all characteristics were analyzed using independent *t*-test for continuous variables and Fisher's exact test for categorical variables. BP, blood pressure; kg, kilogram; KBIT-2, Kaufman Brief Intelligence Test, Second Edition; m, meter; mm Hg, millimeter mercury; MMSE, Mini-Mental State Examination.

2 Independent *t*-test.

3 Fisher's exact test.

## Results

### Participant characteristics

**FIGURE 1:**
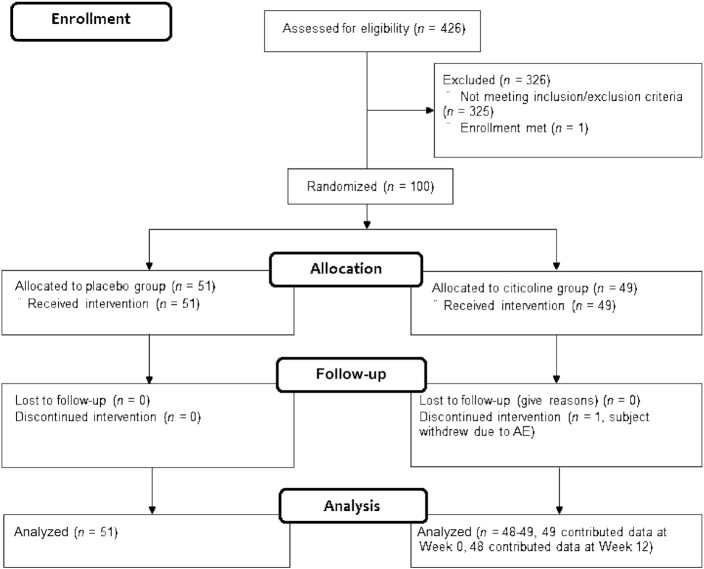
CONSORT participant flow diagram.

A CONSORT diagram illustrating participant recruitment and attrition during the trial is presented in **Figure 1**. A total of 426 participants were screened for participation and 100 participants were randomized. A total of 99 participants completed the study in its entirety. One participant in the citicoline group withdrew from the study due to a headache, which was judged as possibly related to study product. As defined in the study protocol, the ITT sample population consisted of all randomized participants. In the ITT sample population, a total of *n* = 100 participants contributed to data at Week 0 and *n* = 99 participants contributed to data at Week 12. No premature unblinding occurred during the study. There were no differences in selected demographics, and baseline characteristics for sample populations are listed in Table 1. Compliance over the 12-wk supplementation period was 99.2 ± 0.5% for the 99 participants who completed the study.

### Cognitive performance

**FIGURE 2:**
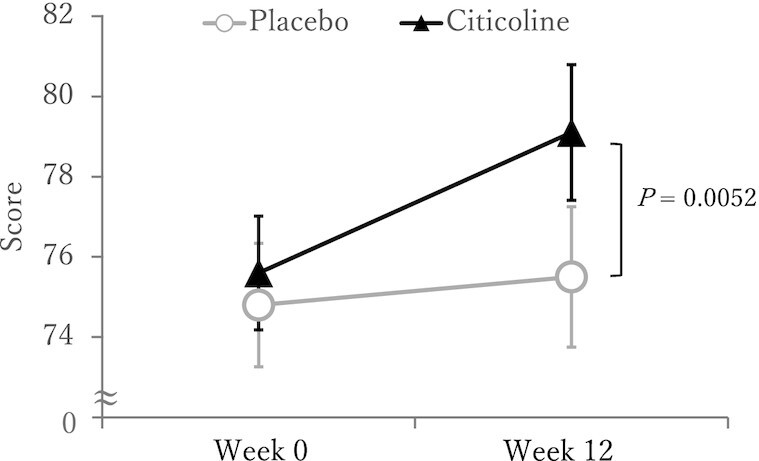
Composite memory scores after 12 wk of citicoline supplementation of the citicoline supplementation group and the placebo group in the ITT population. Participants were healthy older-aged males and females with AAMI. Data shown are unadjusted means ± SEMs for each test group. *n* = 51 for placebo and *n* = 49–48 for citicoline. An increased score indicates improvement. The *P* value shows between-group difference (vs. placebo) for the raw change score using ANCOVA with Bonferroni correction for multiple comparisons. The raw change was calculated as the difference in scores at baseline to the end of the test period for each participant.

**FIGURE 3:**
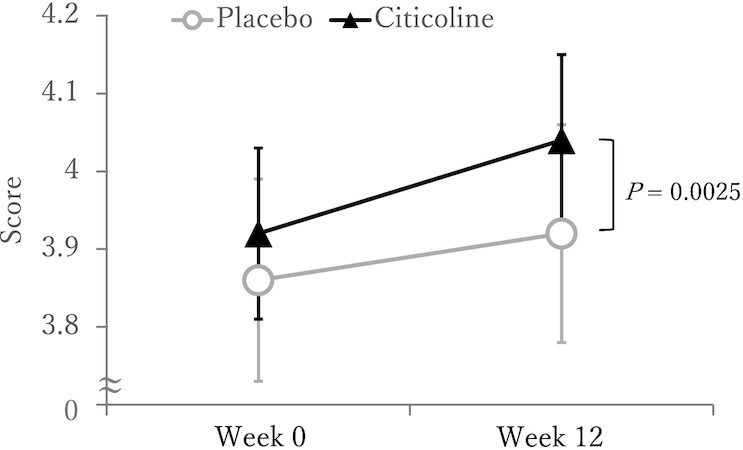
Paired Associates scores after 12 wk of citicoline supplementation of the citicoline supplementation group and the placebo group in the ITT population. Participants were healthy older-aged males and females with AAMI. Data shown are unadjusted means ± SEMs for each test group. *n* = 51 for placebo and *n* = 49–48 for citicoline. An increased score indicates improvement. The *P* value shows between-group difference (vs. placebo) for the raw change score using ANCOVA with Bonferroni correction for multiple comparisons. The raw change was calculated as the difference in scores at baseline to the end of the test period for each participant.

The results on cognitive function tests at Weeks 0 (baseline), and 12 (end of study) are shown in **Table 2**. Within-group analysis indicated that the citicoline group, but not the placebo group, had a statistically significant improvement in Spatial Span, Feature Match, and Composite Memory scores from baseline. Between-group analysis indicated that the changes from baseline scores were statistically significantly different (*P* < 0.00625) between test groups for Paired Associates and Composite Memory whereby the citicoline group demonstrated greater improvements in these tests compared with the placebo group (**Figures** **2** and **3**). No additional statistically significant cognitive effects were detected in Monkey Ladder, Digit Span, Interlocking Polygon Task, and Sustained Attention to Response Task according to multiple comparisons with Bonferroni correction.

**TABLE 2:** Results for cognitive performance of the citicoline supplementation group and the placebo group in the ITT population at Weeks 0 and 12^1^

Test	Baseline (Week 0)	End of study (Week 12)
Spatial Span		
Placebo	4.75 ± 0.11	4.67 ± 0.13
Citicoline	4.65 ± 0.09	4.90 ± 0.12*
Monkey Ladder		
Placebo	6.43 ± 0.12	6.51 ± 0.13
Citicoline	6.65 ± 0.14	6.88 ± 0.17
Digit Span		
Placebo	5.94 ± 0.18	6.22 ± 0.18
Citicoline	5.88 ± 0.16	5.94 ± 0.17
Paired Associate		
Placebo	3.86 ± 0.13	3.92 ± 0.14
Citicoline	3.92 ± 0.11	4.04 ± 0.11†
Composite Memory		
Placebo	74.8 ± 1.5	75.5 ± 1.8
Citicoline	75.6 ± 1.4	79.1 ± 1.7^*†^
Feature Match		
Placebo	83.8 ± 3.0	88.6 ± 3.3
Citicoline	86.7 ± 2.8	96.8 ± 3.5*
Interlocking Polygon Task		
Placebo	26.2 ± 2.3	28.0 ± 2.3
Citicoline	22.8 ± 2.4	25.3 ± 2.8
Sustained Attention to Response Task		
Placebo	174 ± 7	185 ± 2
Citicoline	181 ± 4	177 ± 6

1 Values are means ± SEMs of test score. *n* = 51 for placebo and *n* = 49–48 for citicoline. An increase composite memory score and all of each individual cognitive tests score indicates an improvement. *P < 0.05 within-group difference (baseline vs. end of study) using paired *t*-test. †*P* < 0.05 between-group difference for the raw change score analyzed using ANCOVA with Bonferroni correction for multiple comparisons. The raw change was calculated as the difference in scores at baseline to the end of the test period for each participant.

### Safety assessments

No AEs were judged to be serious. Eight mild AEs were judged to be “possibly” related to 1 of the study products, with 2 occurring in the placebo group and 6 occurring in the citicoline group. All 6 of the AEs in the citicoline group [increased appetite, weight gain, increased flatulence (2 instances), headache, and increased burping] were mild and transient, and were not unexpected based on previous studies with the product (15, 16). No AEs were deemed “definitely” or “probably” related to the ingestion of study product. Data from the hematology and metabolic panels obtained at baseline and end of study are presented in **Supplemental Table 1** and **Supplemental Table 2**, respectively. There were no statistically significant differences in mean values between groups at baseline or at end of study. None of the values fell outside the normal range.

## Discussion

To our knowledge, this is the first randomized, double-blind, placebo-controlled parallel study to evaluate the effects of chronic (12 wk) supplementation of 500 mg/d of citicoline in healthy adults with AAMI on memory performance. Compared with those taking placebo, participants taking citicoline demonstrated a significant improvement in episodic memory assessed using the Paired Associate task and overall memory assessed by the composite memory score. Although each cognitive test assessed distinct components of memory and tapped into different processes, composite score provides for a single outcome variable combining each cognitive test. Our observations are consistent with previous studies demonstrating beneficial effects of citicoline on memory (17, 18).

Episodic memory describes the ability to remember and recall specific events, paired with the content in which they occurred, such as identifying when and where an object was encountered (24). A decline in episodic memory often manifests as the inability to recall past events or retrieve lessons from past experiences, which can lead to repeat error. Episodic memory is more vulnerable than other memory systems to decreases due to aging (25). Kinugawa et al. (26) reported that middle-aged (48–62 y) and aged (71–83 y) participants showed lower episodic memory score as compared with the young (21–45 y) participants with longitudinal studies demonstrating a decline after age 60 y (27, 28). To the best of our knowledge, our study is the first to demonstrate a beneficial effect of citicoline supplementation in maintaining and/or improving episodic memory that may decline with age.

In addition to a statistically significant improvement in Paired Associates, we also observed a tendency toward improvement in the citicoline group for the Spatial Span task. Owen (20) reported activation of the mid-ventrolateral frontal cortex during the Spatial Span and Paired Associates tasks. Citicoline has been shown to improve frontal lobe bioenergetics and alter phospholipid membrane turnover in humans (29). Age-related declines in cognitive abilities, particularly related to function in frontal lobe has been demonstrated in humans (30, 31). Taken together, the findings suggest that activation of the mid-ventrolateral frontal cortex is a possible mechanism of action by which citicoline improved cognition. Future studies may consider assessing regionally specific neuronal activation following citicoline supplementation to better understand its mechanism of action and effect on brain function.

We also observed an improvement in selective attention (assessed using Feature Match task) in the citicoline group compared with baseline; however, there was no significant difference between groups. The primary objective of this study was to assess the effect of citicoline on short-term spatial memory, and thus, is likely underpowered to assess difference in attention between groups. However, the within-group improvement that we observed along with the positive effect reported by a previous study (16), suggests a promising beneficial effect of citicoline on attention that warrants further investigation.

Citicoline is well known to increase the synthesis of phosphatidylcholine, which is the primary phospholipid of neuronal membrane. Studies in rodents have demonstrated increased phosphatidylcholine levels in the brain following repeated citicoline supplementation (32, 33). A clinical study in healthy participants consuming 500 mg/d of citicoline for 6 wk demonstrated increased levels of phosphodiesters, a noninvasive biomarker of phospholipid synthesis in the brain, thus supporting the ability of citicoline to increase brain phosphatidylcholine synthesis in humans (34). Phosphatidylcholine is essential for cell membrane integrity and repair (35, 36, 37), and is normally reduced in brain as a result of aging (38). Plasma phosphatidylcholine levels are positively associated with cognitive flexibility in healthy older adults, and the inferior prefrontal cortex mediates the relationship between plasma phosphatidylcholine and cognitive flexibility (39). Increase in phosphodiesters correlated with improvement on the California Verbal Learning Test, an assessment of verbal learning and memory deficits, in healthy older adults (34). Taken together, these findings suggest that citicoline may slow or prevent AAMI by influencing specific structures within the brain.

Citicoline is naturally present in humans (40), and is a nontoxic material determined by animal toxicology studies (41, 42, 43). In our study, we did not observe any serious adverse events following daily consumption of citicoline for 12 wk. Consistent with our findings, previous clinical trials involving oral citicoline supplementation of Alzheimer's disease patients for 6 wk at a dose of 500 mg/d or for 12 wk at a dose of 1 g/d reported no serious adverse drug reactions (44, 45). Thus, based on our findings and that of others, oral intake of citicoline at amounts up to 1 g/d is safe and well tolerated. Future investigations are required to determine the acute and longer-term effects of citicoline, in addition to the extent to which the beneficial effects of citicoline on memory last following cessation of citicoline supplementation.

The study has some limitations worth noting. Participants were specifically screened for AAMI, and thus, the effects observed may not be generalizable to young adults, and those with cognitive diseases such as dementia and Alzheimer's disease. Disparities in cognitive functioning by race/ethnicity have been suggested by several studies (46, 47). Thus, future studies with a wider range of ethnicity are warranted to understand if the beneficial effects of citicoline on memory observed in our mostly white Caucasian population may be extended to other ethnic and racial groups. In total, there were fewer males than females in this study (35:65 males:females) although effort was made to balance the number of males and females by intervention group to limit any confounding factors due to sex. Additionally, participants were instructed to maintain their habitual diet and lifestyle in an effort to minimize other confounding factors. The day before test visits (Weeks 0 and 12), information on diet intake and sleeping hours was collected to confirm whether participants maintained their habits. Care was taken to ensure that participants completed all cognitive assessments in a supervised environment where lighting and noise were controlled.

Taken together, dietary supplement of citicoline improves overall memory performance, especially episodic memory in healthy males and females with AAMI. The findings suggest that regular consumption of citicoline (Cognizin) may be safe and potentially beneficial against memory loss due to aging.

## Supplementary Material

nxab119_Supplemental_File

## Notes

This research was sponsored by Kyowa Hakko Bio Co., Ltd. This research did not receive any specific grant from funding agencies in the public, commercial, or not-for-profit sectors. Kyowa Hakko Bio Co., Ltd funded the study and participated in study design, but was not involved in the intervention, data collection, and statistical analysis. The views expressed in this manuscript are those of the authors and do not necessarily reflect the position or policy of Kyowa Hakko Bio Co., Ltd.

Author disclosures: EN and FW are current employees of Kyowa Hakko Bio Co., Ltd. EM and KS are current employees of Biofortis Innovation Services. DC is a current employee of Kyowa Hakko USA, Inc.

Supplemental Tables 1 and 2 are available from the “Supplementary data” link in the online posting of the article and from the same link in the online table of contents at https://academic.oup.com/jn.

Abbreviations used: AAMI, age-associated memory impairment; AE, adverse event; CFR, Code of Federal Regulations; GCP, Good Clinical Practices; ITT, intent-to-treat; TMA, trimethylamine; TMAO, trimethylamine N-oxide.
