# Supplementary material for: Citicoline and Memory Function in Healthy Older Adults: A Randomized, Double-Blind, Placebo-Controlled Clinical Trial
Source: J Nutr. 2021 May 12;151(8):2153–60. doi: 10.1093/jn/nxab119 (PMC8349115; doi:10.1093/jn/nxab119)
Supplement: nxab119_Supplemental_File [file nxab119_supplemental_file.pdf]

Effect of Citicoline on Memory Function in Healthy Older Adults: A Randomized, Double-Blind, Placebo-Controlled Clinical Trial. Nakazaki *et al*. Online supplementary material

Supplementary Table 1 Results of hematology panel of the ITT study participants in citicoline supplementation group and the placebo group at weeks 0, and 12 [1]

	Baseline (Week 0)	End of study (Week 12)
	Mean ± SD	Mean ± SD
White Blood Cell (x 10^3/µL)		
Placebo	6.35 ± 1.44	6.25 ± 1.86
Citicoline	6.24 ± 1.63	6.16 ± 1.59
Red Blood Cell (x 10^6/µL)		
Placebo	4.73 ± 0.43	4.65 ± 0.44
Citicoline	4.79 ± 0.46	4.69 ± 0.42
Red Blood Cell Distribution Width (%)		
Placebo	13.8 ± 1.0	13.7 ± 0.9
Citicoline	13.7 ± 0.7	13.7 ± 0.9
Hematocrit (%)		
Placebo	42.4 ± 3.3	41.8 ± 3.4
Citicoline	42.9 ± 3.8	42.1 ± 3.3
Hemoglobin (g/dL)		
Placebo	14.2 ± 1.2	13.9 ± 1.2
Citicoline	14.4 ± 1.3	14.1 ± 1.2
Mean Corpuscular Hemoglobin (pg)		
Placebo	30.0 ± 2.2	30.0 ± 2.2
Citicoline	30.0 ± 1.7	30.0 ± 1.7
Mean Corpuscular Hemoglobin Concentration (g/dL)		
Placebo	33.4 ± 0.7	33.2 ± 0.7
Citicoline	33.4 ± 0.7	33.4 ± 0.7
Mean Corpuscular Volume (fL)		
Placebo	89.9 ± 5.6	90.2 ± 5.6
Citicoline	89.9 ± 4.2	89.9 ± 4.2
Mean Platelet Volume (fL)		
Placebo	9.05 ± 0.75	9.00 ± 0.83
Citicoline	9.03 ± 0.92	9.01 ± 0.97
Platelet (x 10^3/µL)		
Placebo	241 ± 58	240 ± 60
Citicoline	228 ± 48	232 ± 51
Basophil		
(x 10^3/µL)		
Placebo	0.05 ± 0.05	0.05 ± 0.05
Citicoline	0.06 ± 0.06	0.06 ± 0.05
(%)		
Placebo	0.88 ± 0.52	0.86 ± 0.40

Citicoline	0.98 ± 0.66	1.04 ± 0.35
Eosinophil		
(x 10^3/μL)		
Placebo	0.16 ± 0.10	0.21 ± 0.11
Citicoline	0.14 ± 0.11	0.19 ± 0.12
(%)		
Placebo	2.65 ± 1.48	3.53 ± 1.86
Citicoline	2.49 ± 1.98	3.13 ± 2.15
Lymphocyte		
(x 10^3/μL)		
Placebo	1.76 ± 0.49	1.88 ± 0.62
Citicoline	1.74 ± 0.46	1.76 ± 0.44
(%)		
Placebo	28.3 ± 7.0	31.0 ± 7.0
Citicoline	28.9 ± 7.5	29.9 ± 8.3
Monocyte		
(x 10^3/μL)		
Placebo	0.55 ± 0.16	0.58 ± 0.18
Citicoline	0.55 ± 0.17	0.57 ± 0.20
(%)		
Placebo	8.90 ± 2.49	9.61 ± 2.46
Citicoline	9.00 ± 1.76	9.29 ± 1.58
Neutrophil		
(x 10^3/μL)		
Placebo	3.81± 1.22	3.49 ± 1.38
Citicoline	3.75 ± 1.32	3.58 ± 1.31
(%)		
Placebo	59.2 ± 8.5	55.1 ± 8.5
Citicoline	58.9 ± 8.0	57.0 ± 8.5

[1]Values are unadjusted means ± SDs and were measured in fasting whole blood. n = 51 for placebo and n = 49-48 for citicoline. * $P < 0.05$. Continuous measures were compared between groups with the Wilcoxon rank sum test. At each time point, the P values were adjusted for multiple comparisons with the false discovery rate (FDR).

Supplementary Table 2. Results of complete metabolic panel of the ITT study participants in citicoline supplementation group and the placebo group at weeks 0, and 12 [1]

	Baseline (Week 0)	End of study (Week 12)
	Mean ± SD	Mean ± SD
Glucose (mg/dL)		
Placebo	102 ± 9	100 ± 12
Citicoline	102 ± 9	101 ± 12
Sodium (mmol/L)		
Placebo	139 ± 2	139 ± 2
Citicoline	139 ± 2	139 ± 2
Potassium (mmol/L)		
Placebo	4.72 ± 0.56	4.56 ± 0.59
Citicoline	4.47 ± 0.51	4.30 ± 0.53
Chloride (mmol/L)		
Placebo	105 ± 3	105 ± 3
Citicoline	104 ± 3	105 ± 2
CO_2 (mmol/L)		
Placebo	27.6 ± 2.0	27.8 ± 1.8
Citicoline	27.6 ± 1.7	27.5 ± 1.6
Blood Urea Nitrogen (mg/dL)		
Placebo	13.9 ± 3.5	14.5 ± 3.3
Citicoline	13.8 ± 3.7	14.4 ± 3.4
Creatinine (mg/dL)		
Placebo	0.86 ± 0.18	0.91 ± 0.17
Citicoline	0.91 ± 0.19	0.96 ± 0.20
Total Calcium (mg/dL)		
Placebo	9.48 ± 0.31	9.33 ± 0.35
Citicoline	9.35 ± 0.34	9.27 ± 0.35
Alkaline Phosphatase (U/L)		
Placebo	69.7 ± 18.5	69.3 ± 19.1
Citicoline	63.0 ± 16.4	65.5 ± 14.7
Aspartate Aminotransferase (U/L)		
Placebo	26.1 ± 6.1	24.5 ± 5.9
Citicoline	25.6 ± 6.0	25.2 ± 5.9
Alanine Aminotransferase (U/L)		
Placebo	25.1 ± 11.4	23.4 ± 10.2
Citicoline	23.9 ± 9.1	22.2 ± 6.6
Total Bilirubin (mg/dL)		
Placebo	0.78 ± 0.33	0.72 ± 0.29
Citicoline	0.74 ± 0.20	0.73 ± 0.28
Total Protein (g/dL)		
Placebo	6.98 ± 0.41	6.76 ± 0.40
Citicoline	6.88 ± 0.43	6.68 ± 0.45
Albumin (g/dL)		
Placebo	4.10 ± 0.30	3.91 ± 0.25
Citicoline	4.05 ± 0.24	3.87 ± 0.22
Globulin (g/dL)		
Placebo	2.88 ± 0.39	2.84 ± 0.37
Citicoline	2.83 ± 0.41	2.81 ± 0.42

Albumin / Globulin Ratio		
Placebo	1.45 ± 0.26	1.41 ± 0.22
Citicoline	1.46 ± 0.25	1.41 ± 0.22
Anion Gap (mmol/L)		
Placebo	7.29 ± 1.83	6.67 ± 1.44
Citicoline	6.92 ± 1.72	6.81 ± 1.51
Blood Urea Nitrogen /Creatinine Ratio		
Placebo	16.8 ± 5.1	16.3 ± 4.4
Citicoline	15.6 ± 4.6	15.7 ± 4.8
Calculated Osmolality (mOsm/kg)		
Placebo	289 ± 5	289 ± 4
Citicoline	288 ± 4	289 ± 4

[1]Values are unadjusted means ± SDs, and were measured in fasting heparin plasma. $n = 51$ for placebo and $n = 49$-48 for citicoline. * $P < 0.05$. Continuous measures were compared between groups with the Wilcoxon rank sum test. At each time point, the P values were adjusted for multiple comparisons with the false discovery rate (FDR).
